# Killing mechanism of bacteria within multi-species biofilm by blue light

**DOI:** 10.1080/20002297.2019.1628577

**Published:** 2019-06-26

**Authors:** Sharon Shany-Kdoshim, David Polak, Yael Houri-Haddad, Osnat Feuerstein

**Affiliations:** a Department of Periodontology, Hebrew University-Hadassah Faculty of Dental Medicine, Jerusalem, Israel; b Department of Prosthodontics, Hebrew University-Hadassah Faculty of Dental Medicine, Jerusalem, Israel

**Keywords:** Polymicrobial biofilm, blue light, periodontal disease

## Abstract

**Objectives**: The aim of the study was to characterize the immediate and delayed effects of non-coherent blue-light treatment on the composition and viability of an *in vitro* biofilm composed of anaerobic multispecies, as well as the mechanisms involved.

**Methods**: A multispecies biofilm was constructed of *Streptococcus sanguinis, Actinomyces naeslundii, Porphyromonas gingivalis* and *Fusobacterium nucleatum*, test groups were exposed to blue light. The multispecies biofilm was explored with a newly developed method based on flow cytometry and confocal microscopy. The involvement of the paracrine pathway in the phototoxic mechanism was investigated by a crossover of the supernatants between mono-species *P. gingivalis* and *F. nucleatum* biofilms.

**Results**: Blue light led to a reduction of about 50% in the viable pathogenic bacteria *P. gingivalis* and *F. nucleatum*, vs that in the non-exposed biofilm. Biofilm thickness was also reduced by 50%. The phototoxic effect of blue light on mono-species biofilm was observed in *P. gingivalis*, whereas *F. nucleatum* biofilm was unaffected. A lethal effect was obtained when the supernatant of *P. gingivalis* biofilm previously exposed to blue light was added to the *F. nucleatum* biofilm. The effect was circumvented by the addition of reactive oxygen species (ROS) scavengers to the supernatant.

**Conclusion**: Blue-light has an impact on the bacterial composition and viability of the multispecies biofilm. The phototoxic effect of blue light on *P. gingivalis* in biofilm was induced directly and on *F. nucleatum* via ROS mediators of the paracrine pathway. This phenomenon may lead to a novel approach for ‘replacement therapy,’ resulting in a less periodonto-pathogenic biofilm.

The bacteria in the oral cavity are organized in multispecies biofilms [1], which contain about 500–700 taxa at the species level and is the cause of several infectious oral diseases, such as caries and periodontal disease [2,3]. It is well known that bacteria within the biofilm exhibit characteristics different from those under planktonic conditions, due to the expression of specific genes [4]. The bacteria within the biofilm also display resistance to antimicrobial agents, physical forces, nutrient deprivation, pH changes, oxygen radicals and the immune system [5]. The concept of polymicrobial synergy among members of the biofilm is well documented, and experiments consistently show higher pathogenicity of mixed infection compared with monospecies infection [6–8]. This is also attributable to inter-taxa paracrine communication, such as quorum sensing, which facilitates bacterial virulence and survival [9]. Periodontal disease is an infectious, destructive disease characterized by chronic inflammation that eventually leads to tooth loss [10,11]. Previous reports showed that there is a correlation between load of Gram-negative anaerobic bacteria in the sub-gingival sulcus and disease severity [12]. Of those, *Porphyromonas gingivalis* and *Fusobacterium nucleatum* are known keystone pathogens of periodontal diseases, and are often identified together in the subgingival biofilm of periodontal lesions [13–19]. Gram-positive bacteria implicated in periodontitis include oral streptococci of the Mitis group and *Actinomyces naeslundii*. The streptococci once considered strict commensals in the oral cavity [6, 24] do not cause significant pathological changes [6,20,21]. However, mixed infections (for example, with *P. gingivalis*) may result in increased disease severity [6]. These findings contributed to the polymicrobial synergy and dysbiosis hypothesis [6,22], according to which the synergistic polymicrobial community is the cause of periodontitis. Within this community, specific constituents or combinations of functional genes fulfill distinct roles that converge to shape and stabilize a dysbiotic microbiota, which perturbs host homeostasis [6,23].

For almost 100 years the gold-standard treatment for periodontitis has been mechanical subgingival debridement (previously termed scaling and root planning) [24]. However, mechanical debridement does not always lead to major clinical improvement which sometimes can be ameliorated considerably by the adjunctive use of antibiotics [24]. Yet, antibiotics have side effects, and when overused may contribute to antibiotic resistance [20,21,25–27]. An alternative antimicrobial approach to the treatment of periodontitis is lethal photosensitization or antimicrobial photodynamic therapy (aPDT). Visible light, augmented by an exogenous photosensitizer (light-absorbing dye molecules), has been found to be effective as a non-specific treatment against a number of microbial species [28–33], and for inactivation of oral biofilm [33,34]. However, the use of dyes is associated with two main problems: the inability of the dye to diffuse throughout the biofilm and the compromised esthetic results owing to staining of the oral tissues. Despite the development of stain-free Phenalen-1-one photosensitizers (PS), such agents have not been approved for clinical use [21,34].

Having endogenous photosensitizers, black-pigmented bacteria (such as *P. gingivalis*) do not require the addition of dyes for the phototoxic effect [35–38]. Moreover, we previously showed that blue light had a phototoxic effect not only on the periodontal pathogen *P. gingivalis* but also on *F. nucleatum*, both with an impact greater than that on streptococcal species [39]. This selective phototoxic effect on the anaerobic bacteria is probably due to the photochemical mechanism mediated by reactive oxygen species (ROS) formation [40]. Therefore, in the absence of enzymes capable of scavenging ROS, anaerobic bacteria become more sensitive to oxygen when exposed to blue light [40].

Although various studies have demonstrated the selective inhibitory effect of blue light on periodonto-pathogens grown in biofilms *in vitro* and *in vivo* [11,35,41], the phototoxic mechanism of the anaerobic bacteria when in biofilm has been barely explored. As these bacteria are commonly adjacent to each other due to co-aggregation [42,43], we assumed that paracrine signaling may occur between *P. gingivalis* and *F. nucleatum*. Previous investigations of the effect of blue light on *Streptoccocus mutans* biofilm suggested a delayed bacterial death phenomenon, evident 6 h after the biofilm was exposed to light [44,45]. Thus, the objectives of the present study were to characterize the immediate and delayed effects of blue-light treatment on the composition and viability of an *in vitro* anaerobic multi-species biofilm model and to evaluate the possible contribution of bacterial interaction through a paracrine pathway to the phototoxic mechanism.

## Materials and methods

### Bacteria

*F. nucleatum* PK1594, *P. gingivalis* ATCC 33277, *S. sanguinis* NC02863 and *A. naeslundii* 17233 were grown in Wilkins-Chagren broth (Oxoid, Basingstoke, Hampshire, UK), and incubated at 37°C for 24 h under anaerobic conditions (N_2_ 85%, H_2_ 5%, CO_2_ 10%). *S. sanguinis* and *A. naeslundii* were transferred to Wilkins broth enriched with 2% sucrose (Sigma, Rehovot, Israel) and cultured under anaerobic conditions for an additional 24 h. *F. nucleatum* and *P. gingivalis* were transferred to Wilkins broth and incubated for an additional 24 h under anaerobic conditions. The bacteria were then centrifuged (4,000 rpm, 15 min) and suspended in gingival crevicular fluid (GCF)-simulating medium [46] (60% RPMI medium, 40% donor horse serum (Biological Industries, Beit Ha’emek, Israel)) enriched with 5 µg/mL hemin (Sigma) and 0.5 µg/mL menadione (Sigma). The bacterial suspensions of *S. sanguinis, A. naeslundii and P. gingivalis* were adjusted spectrophotometrically to 10^9^ cells/mL, and that of *F. nucleatum* was adjusted to 10^8^ cells/mL [47–50].

### Labeling of specific bacteria in biofilm

To focus on the interaction between specific bacteria within the multi-species biofilm and to examine the effect of blue light on the composition and the viability of each bacterial strain in the biofilm, a novel method was developed that entailed fluorescent labeling of the bacteria and flow cytometry. The assay is based on Fluorescein isothiocyanate (FITC) labeling of a particular bacterial species before its incorporation in the biofilm. Then, after light treatment and fluorescent staining for dead bacteria and dissociation of the mature biofilm into a single bacterium suspension, it is analyzed with flow cytometry (for assay and calibration details, see Polak et al. [51]). When specified, before incorporation in the biofilm, *P. gingivalis* or *F. nucleatum* was stained with FITC by incubating the bacteria for 20 min at room temperature in FITC buffer (1 mg fluorescein isothiocyanate (Sigma Rehovot, Israel) in 500 µl 0.5 M sodium carbonate buffer, pH 9, diluted to a total volume of 10 ml in PBS). Excess stain was removed by three washes with PBS. A previous study confirmed that FITC as a dye does not act as a PS, by the similar results obtained using FITC in FACS assay analysis and CFU counts for bacterial viability of light treated and untreated samples in planktonic suspensions [52].

### Multispecies biofilm

Human saliva (Helsinki board approval HMO052511) diluted 1:4 in double distilled water (DDW) [53] was inoculated onto hydroxyapatite (HA) disks (Clarkson Chromatography Products, South Williamsport, PA) and incubated for 30 min at 37^°^C. The disks were washed with PBS, a suspension of *S. sanguinis* and *A. naeslundii* (1:1 ratio in a total volume of 1,000 µl GCF simulating medium) was inoculated, and they were incubated for 24 h at 37°C under anaerobic conditions. The discs with the newly formed biofilm were then washed with PBS, a suspension of *P. gingivalis* and *F. nucleatum* (1:1 ratio in a total volume of 1,000 µl GCF simulating medium) was inoculated, and they were incubated for an additional 48 h at 37°C under anaerobic conditions. The mature biofilms were washed and reconstituted in PBS (400 µl/disk).

### Monospecies biofilm

Hydroxyapatite discs (Clarkson Chromatography Products) were placed in 24-well plates and inoculated with human saliva (Helsinki board approval HMO052511) diluted 1:4 with DDW [53] for 30 min at 37°C. The disks were washed with PBS, each of the four bacterial suspensions was inoculated separately onto the discs (i.e. one strain/disc in 1000 µl GCF simulating medium total volume), and the discs were incubated for 48 h at 37°C under anaerobic conditions. The mature biofilm was washed and reconstituted in PBS (400 µl/disk).

### Light exposure

A halogen lamp (Belleglass HP, Kerr Inc, Orange, CA) was used for exposure to blue light (wavelength 400–500 nm). The light probe (diameter, 14 mm) was set at a distance of 10 mm from the biofilm sample, resulting in light exposure with a power density of 1.2 W/cm^2^, measured by a power meter (Ophir, Jerusalem, Israel). There was a distance of two wells in the 24-well plate between samples exposed to the light. After the biofilm supernatants were removed, the HA discs were washed, and each biofilm sample was reconstituted in PBS (400 μl in each well of a 24-well plate), and exposed to light for 2 min, equivalent to a fluence of 146 J/cm^2^. Fluence was calculated according to the following formula:

Fluence (J/cm^2^) = Power Density (W/cm^2^)×Irradiation Time (sec)

### Flow cytometry (FACS analysis)

Immediately or 6 h after exposure to blue light, the biofilms were washed with PBS, and stained with 1 µg propidium iodide (Sigma) in 600 µL FACS buffer (0.5 M α-lactose monohydrate (Sigma) in PBS) at room temperature for identification of dead cells. The biofilm was then mechanically dissociated by scraping from the HA, filtered through a cell strainer (70 µm), and analyzed with Accuri C6 flow cytometry (BD Biosciences, San Jose, CA), as described in detail by Polak et al. [51].

### Fluorescence staining and confocal scanning laser microscopy

A confocal scanning fluorescence microscope (Olympus FV300, Tokyo, Japan) with a x 10 lens was used to visualize the distribution of live and dead bacteria throughout the biofilm. The live bacteria were observed after SYTO9 staining (LIVE/DEAD BacLight bacterial viability kit (Molecular Probes, Eugene, OR)) and bacteria with a compromised membrane were seen after staining in the dark at room temperature for 25–30 min with a propidium iodide (PI) solution (1.0 mg PI/mL (Sigma)). Scans through the biofilm were made at 5 µm intervals. An Olympus fluoview ver.3.1 viewer (Olympus, Tokyo, Japan) was used for analysis and image processing.

### Paracrine mechanism

To explore the involvement of the light-induced paracrine pathway, an experimental design was established based on blue-light exposure of mono-species (*P. gingivalis* or *F. nucleatum*) biofilm, and crossover of the supernatant between these biofilms. In addition, the involvement of two main factors – proteases and ROS – that may mediate intercellular effects by a paracrine mechanism following biofilm exposure to blue light was explored. Thus, the supernatants were treated with protease inhibitors or ROS scavengers (vitamin C and catalase) before adding them to the other bacterial biofilm. Scavengers in PBS at final concentrations of 20 U/mL vitamin C or 30 mM catalase were added to the biofilm before light exposure.

### Data analyses

All the experiments were performed in triplicate and repeated at least three times. The data were analyzed with a statistical software package (SigmaStat, Jandel Scientific, San Rafael, CA). One-way-repeated measure analysis of variance (RM ANOVA) was applied to test the significance of the differences between the treated groups. If the results were significant, intergroup differences were tested for significance according to the Student’s t-test and the Bonferroni correction for multiple testing.

## Results

### Immediate effect of blue light on multispecies biofilm

**Figure 1.**
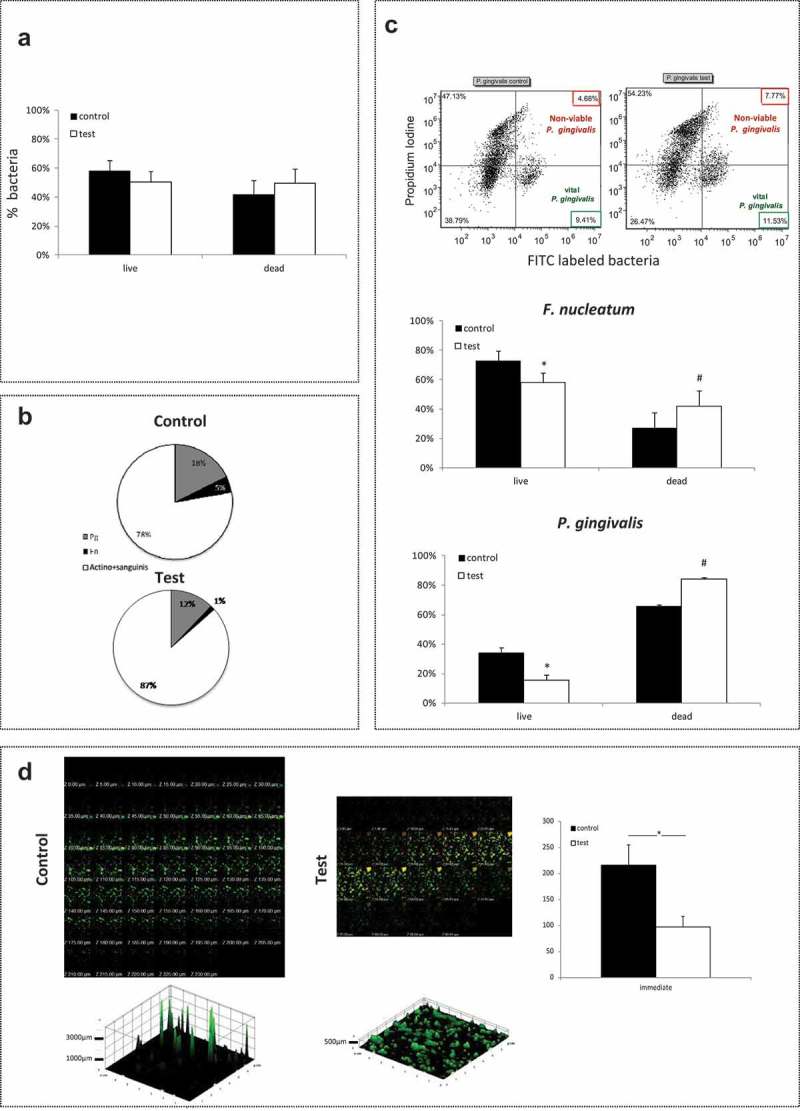
Immediate effect of blue-light exposure on multispecies biofilm Multispecies biofilms was exposed to blue light (test) and compared with non-exposed biofilm (control). *P. gingivalis* (pg) or *F. nucleatum* (fn) was stained with FITC. All the bacteria were stained with the PI marker for dead bacteria. (a) – Percent live bacteria in exposed vs. non-exposed biofilm. (b) – Breakdown of the percentage of viable pathogenic bacteria (pg and fn) in exposed vs. non-exposed biofilm. (c) – Representative dot plots of *P. gingivalis* with FITC and PI staining according to flow cytometry analysis (left – control, right – test), presented as the mean percentages of viable and non-viable bacteria in exposed vs. non-exposed biofilm for *P. gingivalis* and *F. nucleatum*. (d) – Confocal analysis of exposed (right) vs. non-exposed (left) biofilms stained for live/dead, and measurement of biofilm thickness. The results are expressed as the mean ± SD. * and ^#^ significantly different from other groups (live or dead).

Mature, 3-days-old, multispecies biofilms were analyzed with FACS immediately after exposure to light (test group). The results, although not statistically significant, showed that the control multispecies biofilms contained a total (all species included) 60% viable bacteria (Figure 1(a)), whereas the immediate effect of light exposure led to a total 50% viable bacteria (Figure 1(a), test group).

However, exposure to blue light had a clear impact on the composition of the viable bacteria within the biofilm. The relative number of non-pathogenic viable bacteria (*S. sanguinis* and *A. naeslundii*) increased from 78% in the non-exposed biofilm to 87% in the light-exposed biofilm, whereas the relative amount of pathogenic viable bacteria (*P. gingivalis* and *F. nucleatum*) dropped from 23% to 13%, with a reduction in the viability of *P. gingivalis* from 18% to 12% and in that of *F. nucleatum* from 5% to 1% of the total viable bacteria (Figure 1(b)). All the differences were statistically significant (p < 0.05). In addition, the viability percentage calculated for each of the pathogenic bacteria within the biofilm showed a statistically significant reduction immediately after exposure to light, from **≈** 35% to less than 15% for *F. nucleatum*, and from **≈** 80% to **≈** 60% for *P. gingivalis* (Figure 1(c)). Moreover, the biofilm thickness was reduced significantly (p < 0.05) from 220 µm to 100 µm (Figure 1(d)). The confocal images revealed that after exposure to blue light the biofilm contained more dead bacteria than the non-exposed biofilm. This effect was seen in all layers of the biofilm (Figure 1(d)).

## Delayed effect of blue light on multispecies biofilm

**Figure 2.**
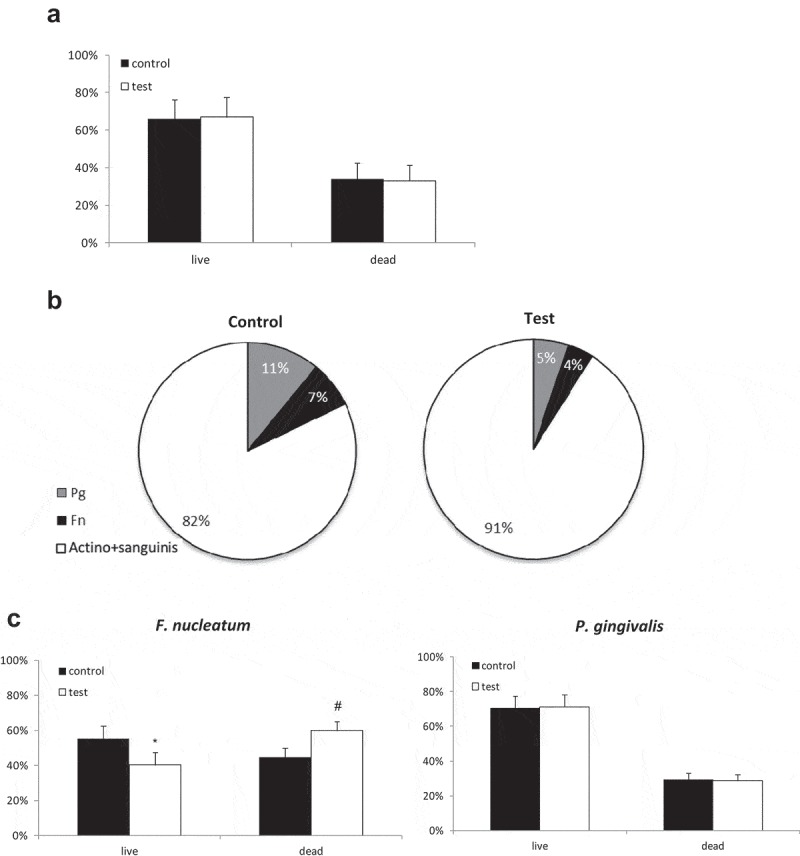
Persistent effect of blue-light exposure on multispecies biofilm. Multispecies biofilm was exposed to blue light and compared with non-exposed biofilm. The biofilm was further incubated for an additional 6 h after exposure. *P. gingivalis* (pg) or *F. nucleatum* (fn) were stained with FITC. All bacteria were stained with the PI marker for dead bacteria. (a) – Percentage of total live and dead bacteria in exposed vs. non-exposed biofilm. (b) – Breakdown of the percentage of viable pathogenic bacteria (pg and fn) in exposed vs. non-exposed biofilm. (c) – Mean percentages of viable and nonviable pg or fn in exposed vs. non-exposed biofilm. The results are expressed as the mean ± SD. * and ^#^ indicate a significant difference from the other groups (live or dead).

Next, the delayed effect was examined after further (6 h) biofilm incubation following light exposure. The multispecies biofilms contained a total of 60% live bacteria (all strains) in both the test and control groups (Figure 2(a)). The shift towards a less pathogenic composition of the biofilm continued 6 h after light exposure, with 9% pathogenic bacteria in the exposed biofilm vs 18% in the non-exposed biofilm, i.e. a reduction of about 50% for each of the pathogenic bacteria (Figure 2(b)). These differences were statistically significant. In addition, the percent viability of each of the pathogenic bacteria within the biofilm showed a statistically significant reduction in the viability of *F. nucleatum* 6 h after exposure to light, from **≈** 60% to **≈** 40%. There was no noticeable difference in the viability of *P. gingivalis* (Figure 2(c)).

## Blue light effect on mono-species biofilm

**Figure 3.**
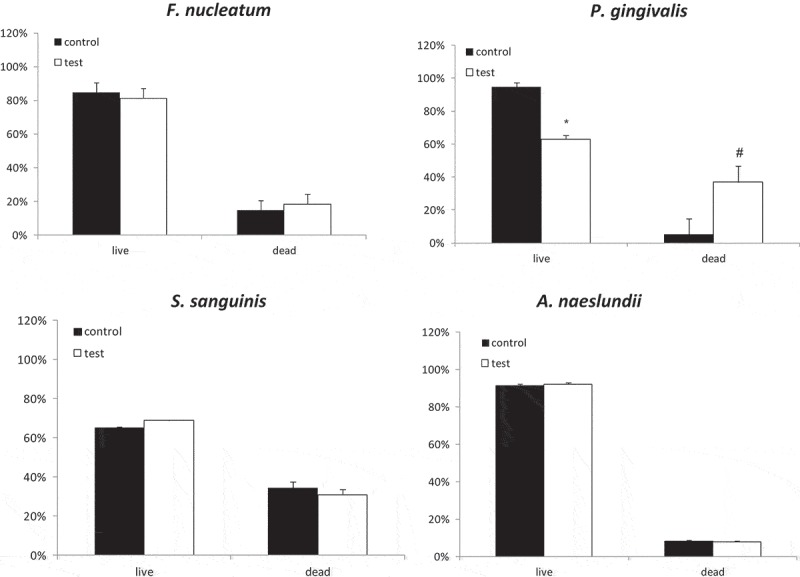
Blue light effect on single species biofilm Mono-species biofilm was exposed to blue light (test) and compared with non-exposed biofilm (control). All biofilms were stained with the PI marker for dead bacteria. The results are expressed as the mean ± SD. * and ^#^ = significant difference from the other groups.

To elucidate the effect of blue light on the multi-species biofilm, its effect on mono-species biofilm formed by each of the four bacteria that composed the multi-species biofilm was examined. Only *P. gingivalis* biofilm exhibited susceptibility to blue light, with a statistically significant reduction in viability from 95% to 60% (Figure 3). Blue light did not affect the other monospecies biofilms (*F. nucleatum, S. sanguinis* and *A. naeslundii)* under the experimental conditions of this study (Figure 3).

## Light-induced paracrine killing mechanism of bacteria

**Figure 4.**
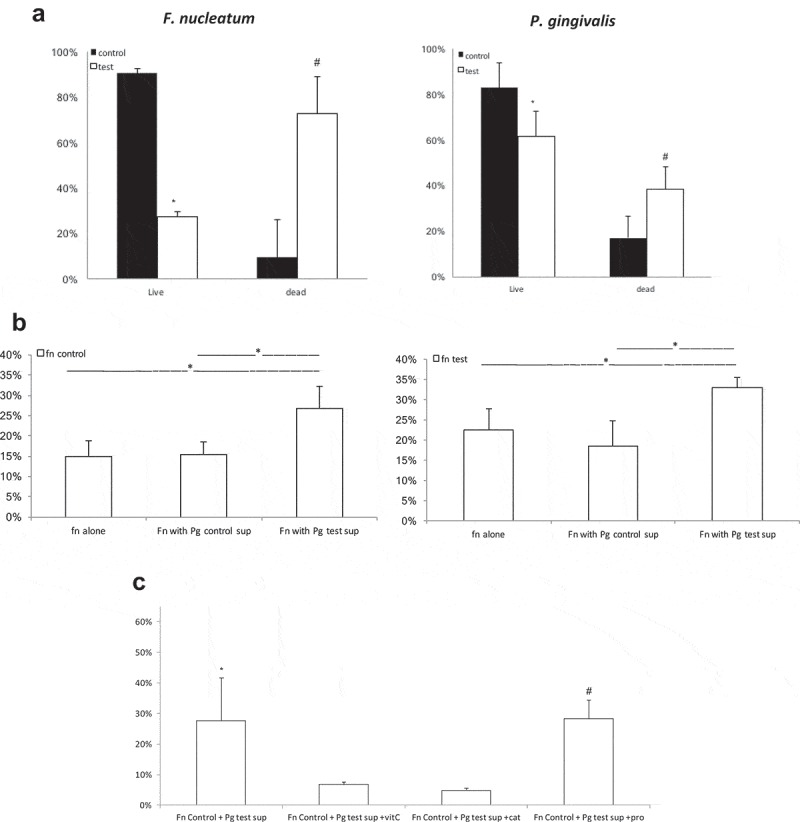
Supernatant paracrine effect of *P. gingivalis* and *F. nucleatum* monospecies biofilms after exposure to blue light. (a) – *P. gingivalis* and *F. nucleatum* biofilms exposed to blue light (test group) or non-exposed (control group) after a supernatant exchange of the two biofilms. (b) – *F. nucleatum* biofilm, non-exposed and exposed to blue light, after the addition of supernatants of *P. gingivalis* biofilm (exposed or non-exposed) and without supernatant (control). (c) – *F. nucleatum* biofilm non-exposed to light, with the addition of supernatant of exposed *P. gingivalis* biofilm, alone and with ROS scavengers (vitamin C or catalase) or protease inhibitor. The results are expressed as the mean ± SD. * and ^#^ = significant difference from the other groups.

To explore the possibility of a light-induced paracrine pathway, an experimental design was established based on blue-light exposure of mono-species (*P. gingivalis* or *F. nucleatum*) biofilm, and crossover of the supernatant between the two biofilms (exposed biofilm supernatant exchange with exposed biofilm). Crossover of the non-exposed biofilm supernatants did not change the viability of *F. nucleatum* or *P. gingivalis* (compare control groups in Figures 4(a) and 3). Light-exposed *P. gingivalis* biofilm introduced into the supernatant of exposed *F. nucleatum* biofilm showed an effect similar to that seen in the previous experiments (reduction of live *P. gingivalis* – Figure 4(a), *P. gingivalis* test group vs that in Figure 3). Light-exposed *F. nucleatum* biofilm introduced into the supernatant of exposed *P. gingivalis* biofilm showed an increased bactericidal effect compared with that seen in the previous mono-specious experiment (Figure 4(a), *F. nucleatum* test group vs that in Figure 3). However, the bactericidal effect was similar to that observed in the multispecies biofilm experiments (Figure 4(a), *F. nucleatum* test group, compared with that in Figure 1(c)). These differences were statistically significant (p < 0.05).

These results show that the bactericidal effect of blue light on *F. nucleatum* biofilm is mediated by a factor present in the *P. gingivalis* supernatant. To determine whether the exposure of *F. nucleatum* to blue light has any impact, an experiment was performed in which non-exposed *F. nucleatum* mono-species biofilm (Figure 4(b), *F. nucleatum* control groups) was compared with light-exposed *F. nucleatum* biofilm (Figure 4(b), *F. nucleatum* test groups). *F. nucleatum* biofilm was introduced into the supernatant of *P. gingivalis* biofilm previously exposed to blue light (as well as to the supernatant of non-exposed *P. gingivalis* biofilm) and was compared with *F. nucleatum* biofilm with its own supernatant. The paracrine bactericidal effect was observed only when the supernatant of *P. gingivalis* biofilm previously exposed to blue light was used, regardless of *F. nucleatum* biofilm exposure to blue light (Figure 4(b)).

Two main factors may mediate the destructive interaction between *P. gingivalis* supernatant (following exposure to blue light) and *F. nucleatum* biofilm – (a) robust expression of *P. gingivalis* proteases, which may induce bacterial lysis, (b) expression of reactive oxygen species by *P. gingivalis* following exposure to blue light, which affects *F. nucleatum* viability. To determine the source of this interaction, the *P. gingivalis* supernatant was treated with protease inhibitors or scavenger molecules (vitamin C or catalase) before the crossover with *F. nucleatum* biofilm. The results showed that in the presence of ROS scavengers the *P. gingivalis* supernatant had a reduced bactericidal effect on the *F. nucleatum* biofilm, whereas supernatant with or without the addition of protease inhibitor had a similar bactericidal effect on the *F. nucleatum* biofilm (Figure 4(c)).

## Discussion

The current study shows that blue light has an impact on the bacterial composition and viability of the multispecies biofilm. The phototoxic effect of blue light on *P. gingivalis* in biofilm was induced directly and on *F. nucleatum* via ROS mediators of the paracrine pathway. This phenomenon may lead to a novel approach for ‘replacement therapy,’ resulting in a less periodonto-pathogenic biofilm.

The present investigation aimed to examine not only the immediate but also the late effect of blue light on the composition of viable bacteria in the multi-species biofilm. As the simulation of the complex structure of the oral biofilm was beyond the scope of this study, an *in vitro* model simulating a periodontitis-associated dysbiotic biofilm, based on keystone pathogens of periodontal diseases, was established. The biofilm was grown on surfaces similar to those of the tooth (HA) and in a medium simulating the natural environment of the periodontal pocket [46]. Although real-time PCR (qPCR) is a widely used technique in quantitative analysis of multi-species samples, its main limitation is its inability to discriminate between live and dead cells. This problem was overcome by the use of a unique and reproducible assay developed to quantify dynamic changes in the viability of specific species in the biofilm with the aid of fluorescent staining and flow cytometry. Similarly, Cieplik et al. [21] assessed the damage of cytoplasmic membranes of three different bacteria in the biofilm. However that study was performed on a non-fluorescent side scatter FACS scale, which measures granularity and not always can be used to differentiate between different bacteria. The novelty of the present study is the ability to clearly differentiate fluorescently between the target pathogen and the other bacteria in a multispecies biofilm by using a flow cytometry setting.

This study demonstrates the selective bactericidal effect of blue light on the growth of *P. gingivalis* and *F. nucleatum*. Light exposure (at a fluence of 146 J/cm^2^) led to a **≈**50% reduction in the relative number of viable pathogenic bacteria. Since this change in biofilm composition involves keystone pathogens, makes it highly significant in microbiome dysbiosis processes. This relative change in the bacterial composition of the biofilm was maintained for at least 6 h after exposure. In addition, blue light led to a reduction in total biofilm thickness by **≈** 50%, indicating an additional anti-biofilm effect of the light, which could be due to its direct or indirect continuous effect on bacterial interaction. Previous studies showed the long-term effect of light on the previously exposed *Streptococcus mutans* in biofilm, which could be due to the delayed death phenomenon of bacteria and/or as a result of changes in gene expression [44,45]. Thus, other mechanisms, such as co-aggregation, metabolic communication and quorum sensing [54], aside from the direct phototoxic effect of the light on the keystone periodonto-pathogens, may be involved and continue to affect the whole biofilm. These results correlate for the most part with those of Fontana et al. who showed a 50% reduction in bacterial growth on blood agar following blue light exposure, with a reduction in the percentage of the periodonto-pathogenic bacteria in the biofilm [11]. The phenomenon of selective reduction by phototargeting of human periodontal pathogens, such as black-pigmented species and *F. nucleatum*, was also demonstrated *in vivo* by twice daily application of blue light to oral plaque over a period of 4 days [41].

In the present *in vitro* model, as the composition and conditions of biofilm growth and exposure to light were controlled, it was possible to compare the effect of blue light on the multispecies biofilm with that on a specific mono-species biofilm of each bacterial strain within the biofilm, under similar conditions. Blue light affected only *P. gingivalis* biofilm, without any significant impact on *F. nucleatum* biofilm, although the exposure affected both bacteria when in a multispecies biofilm. These findings do not correlate completely with our previous results showing the high susceptibility to blue light of the two strains, when exposed in the planktonic state or when on agar plates [39]. This could be explained by the differences in *F. nucleatum* susceptibility to light when in the planktonic state, on agar or as a biofilm. Indeed, the above results were in agreement with those of Song et al. who found that after exposure to blue light, a similar reduction in *P. gingivalis* viability was observed in both the planktonic and biofilm states, whereas in *F. nucleatum* this was seen only in planktonic bacteria [35]. Another study suggested that *F. nucleatum* plays a protective role against ROS, atmospheric O_2_ and H_2_O_2_ within the oral biofilm, owing to protein defense systems [52], which may account, as previously demonstrated for the enhanced tolerance of *F. nucleatum* to blue light when in mono-species biofilm.

On the other hand, it was well demonstrated that, compared with streptococcal species, anaerobic bacteria, such as *P. gingivalis* and *F. nucleatum*, were more susceptible to blue light under aerobic conditions, owing to their greater sensitivity to oxidative stress [40]. The mechanism by which black-pigmented oral bacteria such as *P. gingivalis* were sensitized by blue light was attributed to their accumulation of iron protoporphyrin IX (PpIX), as endogenous photosynthesizer [35,55]. However, another study showed that although the amounts of endogenous porphyrin produced by *Prevotella* species were greater than those produced by *Fusobacterium* species, both genera exhibited a similar susceptibility to blue light [56]. Thus, it appears that photo-targeting of other endogenous chromophores, such as cytochromes and flavins, may contribute to the increased phototoxicity of fusobacteria [40], as well as to that of other periodontal pathogens, such as *Aggregatibacter actinomycetemcomitans* [57]. As *F. nucleatum* and *P. gingivalis* co-aggregate [58] and are probably located close to each other within the biofilm, we postulated that ROS production and/or the enzymatic content of *P. gingivalis* dead cells may affect the viability of *F. nucleatum* when in multi-species biofilm. Indeed, the results show that *F. nucleatum* in biofilm was not affected directly by blue light. However, the supernatant of *P. gingivalis* previously exposed to blue light had an impact on viability, and this effect was found to be mediated by oxidative species. Therefore, it is reasonable to assume that the phototoxic effect of blue light on *F. nucleatum* when in multi-species biofilm may not be a direct effect but mainly an indirect effect mediated by ROS and resulting in the reaction of *P. gingivalis* to the light.

The present study confirms the notion that the anaerobic pathogens *P. gingivalis* and *F. nucleatum* are more susceptible to the oxidative stress resulting from the blue-light effect on bacteria or via ROS produced by neighboring affected bacteria, than the commensal aerobic bacteria within the biofilm. Moreover, the exposure of biofilm to blue light induced a shift, maintained for at least 6 h, towards a less pathogenic composition as well as total biofilm reduction. The investigation demonstrates that blue light may serve as a potential tool for facilitating a sustained shift from a dysbiotic periodontal biofilm to a symbiotic biofilm. However, as the conclusions obtained here are derived from *in vitro* studies, their relevance to the processes occurring *in vivo* and the appropriate clinical protocol with the precise conditions of the light therapy should be further investigated. Yet, it appears that the blue-light technique might be applicable in periodontal disease, and may represent a novel approach to ‘replacement therapy’ in other biofilm-associated diseases.
